# A Qualitative Experimental Proof of Principle of Self-Assembly in 3D Printed Microchannels towards Embedded Wiring in Biofuel Cells

**DOI:** 10.3390/mi14040807

**Published:** 2023-03-31

**Authors:** Terak Hornik, James Kempa, Jeffrey Catterlin, Emil Kartalov

**Affiliations:** Naval Postgraduate School, Monterey, CA 93943, USA

**Keywords:** 3D printing, additive manufacturing, embedded, wiring, microchannel, biofuel, cell, electric, conductive, microfluidic, self-assembly, experimental

## Abstract

A range of biotech applications, e.g., microfluidic benthic biofuel cells, require devices with the simultaneous capabilities of embedded electrical wiring, aqueous fluidic access, 3D arrays, biocompatibility, and affordable upscalability. These are very challenging to satisfy simultaneously. As a potential solution, herein we present a qualitative experimental proof of principle of a novel self-assembly technique in 3D printed microfluidics towards embedded wiring combined with fluidic access. Our technique uses surface tension, viscous flow, microchannel geometries, and hydrophobic/hydrophilic interactions to produce self-assembly of two immiscible fluids along the length of the same 3D printed microfluidic channel. The technique demonstrates a major step towards the affordable upscaling of microfluidic biofuel cells through 3D printing. The technique would be of high utility to any application that simultaneously requires distributed wiring and fluidic access inside 3D printed devices.

## 1. Introduction

Microbial fuel cells (MFC) use bacteria as a tool to oxidize organic matter with the intent of producing electricity [1,2,3,4]. A subclass of MFCs are benthic microbial fuel cells (BMFC) [1,3,4,5,6], which extract power from electrons expelled by bacteria that inhabit the ocean floor ecosystems [7]. Reported results [1] show ranges of 0.2–1 V for the output voltage and 3–40 mW/m^2^ for the power density. As a result, BMFCs generally find their niche with applications involving low power devices, whose locations make other power sources impractical. Examples include, but are not limited to, meteorological buoys [5], autonomous sensors/acoustic modems [6], and magnetometers for detecting the presence of ships [8].

Macroscale BMFCs have macroscale separation between bacteria and capture electrodes. As a result, it is reasonable to expect that most electrons are not captured, which diminishes the power output of the biofuel cell. It directly follows that shortening the average electron travel distance would increase the electron capture efficacy and increase power output. We recently reported [1] on microfluidic benthic microbial fuel cells (MBMFC) that minimize the distance between the electron-emitting bacteria and the capture anode from typically centimeters to less than 100 μm. Consequently, significantly increased power densities [1] (80 mW/m^2^) were measured.

These MBMFCs [1] relied on the assembly of a substrate bearing the electrode network to a replica-molded microfluidic element bearing a two-dimensional array of microchannels. The resulting chip [1] was excellent as an experimental proof of concept for the MBMFCs. However, such 2D structures cannot be affordably fabricated and assembled into a 3D array big enough to produce a practically useful amount of electrical power. Overcoming these limitations is the next step in the development of MBMFCs as a renewable source of electrical power.

3D printing is an ideal candidate to address these limitations. It would be optimal for the upscaling of such a device because of its repeatability, scalability, streamlined automated manufacturing, and prototyping speed. 3D printing also offers inbuilt integration, thereby avoiding the cost of manual assembly of many identical subsystems, which would otherwise be needed due to the relatively low power densities of BMFCs [1,2,3,4,5]. This improvement alone has the potential to make the overall macro-scale system commercially viable.

The switch to 3D printing-based fabrication methodology introduces two new roadblocks. First is the removal of sacrificial material from inside the microchannels, which is a general problem in any 3D printed embedded microfluidics. We recently reported [9] on a successful solution to this problem.

The second roadblock is the construction of the anode wiring. One solution to this is to use multi-material 3D printing to form the embedded wires. This has already been attempted by fused filament fabrication [10], and combined DLP and SLA printing [11]. Fused filament fabrication is a promising idea, but its 0.4 mm diameter minimum filament size [10] makes it of very limited use to microfluidic or other microscale applications that require smaller features. Another approach [11] is to interrupt a 3D print to embed a circuit, prior to finishing the print. However, this leads to higher expense, bonding issues, introduction of stress concentrations, decreased automation, and increased difficulty in upscaling. A third alternative is multi-material digital light processing 3D printing (MM-DLP3DP), which offers improved surface finishes and resolution, and is overall a promising fabrication methodology. However, it is inapplicable inside microchannels due to the increased difficulties in surface preparation and in the metal deposition process itself [12]. In addition, palladium has limited biocompatibility [13], so would likely not work well with MBMFCs. Finally, palladium is a costly and rare material which would challenge the commercial affordability. A fourth alternative solution is to construct wires inside already printed structures, e.g., by self-assembly techniques [14,15,16,17]. Herein, we report on one such self-assembly method.

This method uses microchannels, whose cross-section has a tall wide center flanked by two shallow flanges. The hydrophobic microchannel is filled with hydrophobic fluid, which is then displaced by a hydrophilic fluid. Surface tension ensures that the hydrophobic fluid is retained inside the flanges, while the center is cleared. This self-assembly would work along the entire length of the channel, and thus should work in any architecture made of such individual microchannels. An experimental proof of principle for the self-assembly technique is presented herein.

The experimental results indicate that embedded wires would self-assemble under the proper conditions, allowing for 3D printing to be used to fabricate MBMFCs [1]. More generally, the same technique should be useful in any application requiring electrically conductive wiring and fluidic access inside 3D printed devices.

## 2. Materials and Methods

**Figure 1:**
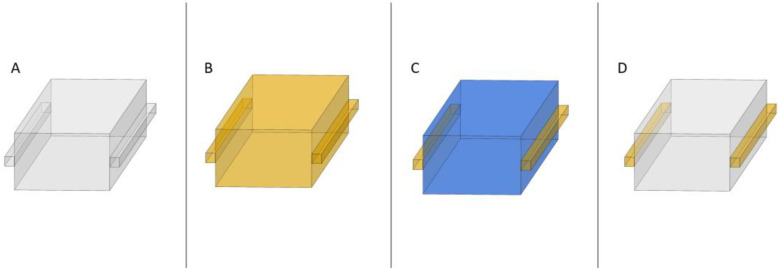
Self-Assembly Scheme. A flanged microchannel (**A**) formed as a cavity in a 3D printed chip in hydrophobic resin is filled with hydrophobic liquid (**B**). Flushing hydrophilic liquid at moderate pressure would clear the central section of the channel but leave the flanges filled with the hydrophobic liquid (**C**). This self-assembles the two immiscible liquids using the same channel. Any structure made of this basic channel should benefit from the same scheme. If desired, the hydrophilic liquid can be later removed by flushing air (**D**). If desired, the hydrophobic fluid in the flanges may be thermally or optically cured in position. If the hydrophobic fluid is electrically conductive, the described scheme simultaneously offers embedded wiring and aqueous fluidic access, e.g., to the benefit of a range of biotech applications.

**Figure 2:**
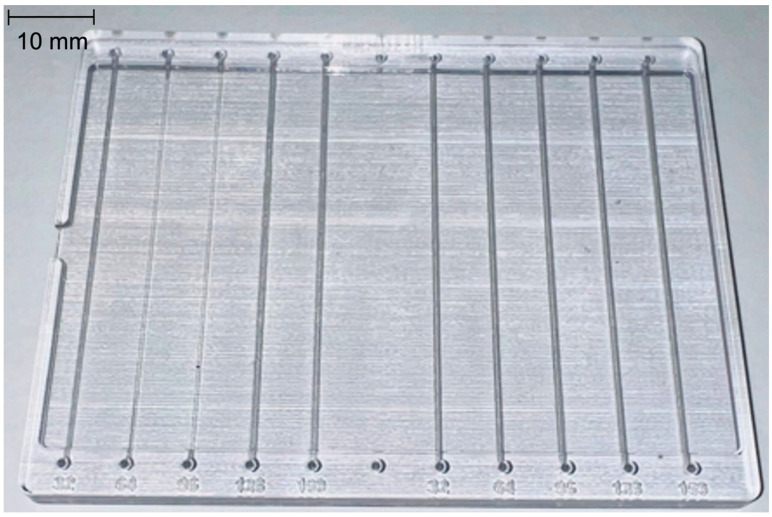
Test chip architecture. Flanged channels were arranged in 2 groups of 5 channels, with central sections’ planned dimensions of 192 µm (**left**) and 288 µm (**right**), respectively. Within each group, the planned dimensions of the flanges were varied as follows: 32, 64, 96, 128, 160 µm square. Actual printed dimensions were larger due to a known widening phenomenon [9].

*Chip Architecture.* Each microchannel had a cross-section design, including a square central part and two square flanges (Figure 1). The microchannels were built in test chips designed as a rectangular prism (90 × 69 × 4 mm) (Figure 2), wherein the top surface of the chip had a raised rim to enclose a layer of mineral oil, which would smoothen the surface and improve optical clarity when imaging. For each chip, the inlet/outlet ports were designed to allow a 23-gauge luer stub adapter to be held snugly without jamming or leaking. Each chip contained 2 groups of 5 microchannels each. One of the groups had a cross-section with its central part planned as 192 µm square. The other group had a cross-section with its central part planned as 288 µm square. Within each group of microchannels, the square flange size parameter was planned to vary as 32, 64, 96, 128, 160 µm. The dimensions of the large central section were chosen because they were the smallest planned sizes that consistently met with success with our clearing procedure [9]. The smaller flange section’s dimensions were chosen as natural multiples of the advertised resolution of the printer.

*Fabrication*. The test chips were modeled in SOLIDWORKS 2021 (SolidWorks Corp., Waltham, MA, USA) for use with the 3D printer, Stratasys Objet500 Connex 1 (Stratasys Ltd., Rehovot, Israel). The models were then prepared for digital slicing by being positioned flat on the print bed and oriented to make the regular defects inherent in the printing processes orthogonal to the channels, to avoid potential visual distortions. The Objet500 printed the chips monolithically, with SUP706B sacrificial material embedded inside the VeroClear-RGD810 clear resin in a single process. The VeroClear material was selected due to its optical clarity, chemical resistance, and rigidity.

*Clearance procedure.* The previously published protocol [9] was applied to the chips. Briefly, it involves sonication in 2% NaOH aqueous solution, heating at 80 °C, and flushing the microchannels with 10% NaOH aqueous solution.

**Figure 3:**
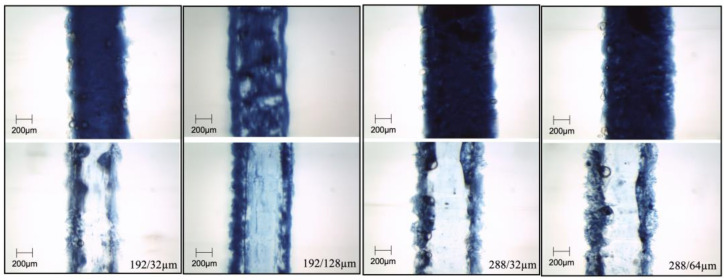
Successful self-assembly. Each pair of images shows the same channel before (**top**) and after (**bottom**) the water flush. The numbers in the bottom right corner of each pair indicate the planned size of the center/flanges. The results demonstrate the successful self-assembly scheme as the flanges remain filled with oil, while the central section of the channels is filled with water.

**Figure 4:**
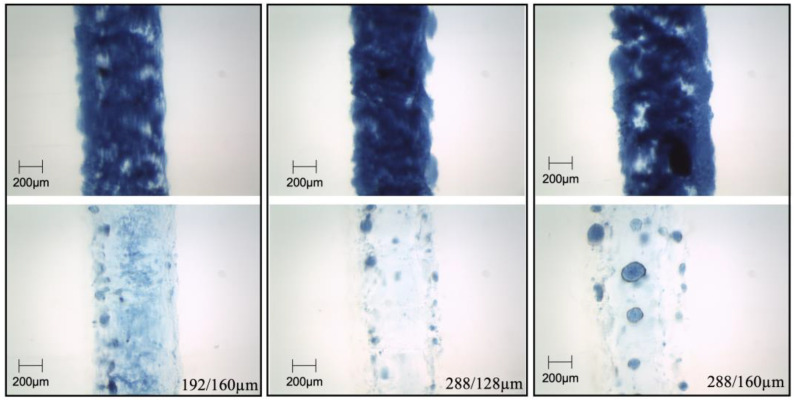
Failed Self-Assembly. Each pair of images shows the same channel before (**top**) and after (**bottom**) the water flush. The numbers in the bottom right corner of each pair indicate the planned size of the center/flanges.

*Chip testing.* After the sacrificial material was cleared out of the chips, mineral oil (J217-500ML, VWR Life Science) was mixed with Prussian Blue oil-based paint (Winsor & Newton, London, UK) in a weight ratio of 5.42:1. The mixture was fed into the microchannels through the ports by 23-gauge luer stub adapters and 1 mL syringes (Becton-Dickinson, Franklin Lakes, NJ, USA). Optical images were taken (top photos in Figure 3 and Figure 4). Next, water was fed in the same way at moderate pressure and then further optical images were taken (bottom photos in Figure 3 and Figure 4).

*Optical setup and imaging.* Optical imaging was performed with a binocular stereo 3.5–90× zoom microscope system (SM-1TZ-PL-10MA, AmScope, United Scope LLC, Irvine, CA, USA). Mineral oil (J217-500ML, VWR Life Science) was applied to the depression in the top surface, to optically smooth out the surface roughness inherent in the printing process. Multiple images from the same channel for the size for each chip were taken at consistent maximal magnification using the 10 MP color camera. A 10 mm calibration slide with 10 µm spacing between its bars was imaged at the same maximal magnification. This image was then used to calibrate the AmScope inbuilt software measurement tool.

## 3. Results and Discussion

3D printing offers many advantages to a wide range of applications: rapid prototyping, inbuilt integration, automated manufacturing, and relatively simple upscalability. However, 3D printing still has some limitations.

First, the challenge of removing sacrificial material from inside 3D printed chips stands in the way of microfluidic applications requiring 3D arrays of sub-devices. We recently reported on a potential solution [9] to this problem involving a clearance protocol that heats, dissolves, and flushes out the sacrificial material using a solution of NaOH. This technique enables the formation of embedded wiring inside 3D printed chips by simply filling the microchannels with conductive fluid to turn them into wiring. This has important implications for a wide range of devices, e.g., artificial muscles [18], microfluidic-based radio frequency identification devices [19], and compact hierarchical manifold microchannel heat sink arrays [20].

Second, the unavailability of conductive/nonconductive hybrid 3D printing stands in the way of 3D printed chips that have requirements for both a fluidic access and an electrical connection to each element of the three-dimensional array as required by a range of applications, e.g., microfluidic benthic micro-bacterial biofuel cells [1]. Herein, we present a potential solution to this second problem.

As noted above, the cleared microchannels may be used to distribute conductive material and thus embed electrical wiring post-print. However, the same microchannels must also be used to load the cells in a continuously flowing aqueous solution. Hence, some form of self-assembly would be required to meet both criteria. A common method of self-assembly in microfluidic applications is to use surface tension between immiscible fluids, e.g., for packaging aqueous material in water droplets in oil [15,16,17]. Hence, we combined these ideas in the self-assembly scheme presented in Figure 1.

In the proposed scheme, a microchannel has a cross-section containing a large central square plus two smaller square flanges on the sides (Figure 1A), after the removal of the sacrificial material. A conductive hydrophobic fluid is fed into the channel and fills it completely (Figure 1B). As the material of the microchannel walls is 3D printed with hydrophobic resin, the hydrophobic liquid will wet the walls, the filling will be very efficient, and there should be no air bubbles remaining. Next, a hydrophilic liquid, e.g., water, is fed into the channel. Small dimensions would produce low Reynolds viscous flow, governed primarily by Poiseuille’s law. Hence, the fluidic resistance in the central large square section would be far smaller than the resistance in the flanges. Therefore, at moderate pressure, the water would shunt through and clear the central square, but would not clear the side flanges. As a result, the side flanges would remain filled with the hydrophobic conductive fluid, while the central section would be filled with water (Figure 1C). The result is what is needed in biofuel cells [1]—electrical wiring reaching out to every volume containing power-generating bacterial cells.

If desired, a final feed of air at moderate pressure could displace and dry out the water, leaving only the hydrophobic conductive fluid behind in the flanges (Figure 1D). At moderate pressure, air should not be able to displace the hydrophobic liquid from the flanges due to surface tension and high wetting between the hydrophobic fluid and the hydrophobic resin walls of the microchannel. If desired, the hydrophobic liquid in the flanges could also be thermally cured or photo-cured for mechanical robustness.

While targeting an experimental implementation of the above scheme, we noted that a self-assembly method that works everywhere in a basic channel should work everywhere in any complex microfluidic architecture that is put together using segments of the same basic channel. Hence, we only needed to show successful self-assembly in that basic channel, to infer success within any complex architecture. Therefore, we designed a 3D printed chip containing just such channels, wherein the flanges were positioned on either side of the channel in the horizontal plane (Figure 1). This was done to facilitate optical imaging from above or below, as well as to simplify the interpretation of the results. However, it is worth noting that other options are also available: (a) having a single flange on one side of the channel only; (b) having one flange on each of the four sides of the main channel; and (c) having multiple flanges on the same side (e.g., for particularly large central channels).

To make efficient use of resources and enable parameter sweeps, we designed the chip with 10 channels organized into two groups of five (Figure 2). Within each group, the large central squares had the same size, while the size of the square flanges was varied consistently. This efficiently provided us with a range of combinations of both parameters. Then, the chip model was 3D printed in three copies. The actual achieved dimensions were wider than planned, due to a 3D printing phenomenon described previously [9]. The chips underwent the clearance protocol [9]. The actual size of the central section ranged from approximately 400 to 800 µm, and the flanges size ranged from approximately 100 to 200 µm.

Mineral oil was mixed with blue oil-based paint and fed into the channels. Optical images were then taken at various locations for all channels on all chips, producing images of filled channels, as shown in Figure 1B. Then, water was flushed through the channels with the goal to obtain the state in Figure 1C, and the result was recorded by optical images. The results are presented in Figure 3 and Figure 4, wherein the images are organized in pairs by channel, before and after the water flush (tops and bottoms, respectively). The flanges can be discerned by boundary lines, e.g., in the first and second pair of Figure 3, and/or as displaying a lighter blue color due to less depth and less absorption compared to the deeper center, e.g., in the top image of the third pair in Figure 4.

Figure 3 shows examples of successful self-assembly. Critically, the blue paint in oil mixture is retained in the flanges but removed from the central section of the channels. In contrast, Figure 4 shows examples of failed self-assembly. The general observation is that the self-assembly depends on both the applied pressure and the dimensions of the channel. If the pressure is high enough, the water will clear the flanges as well. Larger center and flange dimensions also correlate with self-assembly failure. Empirically, it appears that the cutoff is around 500/150 µm size of center/flanges. Overall, the results offer an experimental qualitative proof of principle for the self-assembly technique in 3D printed microfluidics.

Future work would optimize the conditions and calibrate applied pressure vs dimensions to determine the cutoff pressure for each dimension combination. Separately, the dye in oil mixture would be replaced with conductive hydrophobic liquid so that the wiring is experimentally demonstrated, and the resulting channel’s electrical conductance can be quantified. Conductive non-metallic particles suspended in a prepolymer carrier liquid that is fed in, self-assembled, and then (optically or thermally) cured in position, may provide the best solution for the desired properties in biofuel cells [1] and similar biotech applications. The use of metal emulsions for this application would be counterproductive due to (a varying degree of) toxicity to the cells, but may be beneficial in other applications. Overall, the presented technique and the charted future work should lead to important new capabilities, particularly in biotech and renewable energy.

## 4. Conclusions

The presented work is a qualitative experimental proof of principle of a novel technique for the self-assembly of wiring structures embedded in microchannels in 3D printed microfluidics. Our technique, relying on the cross-section geometries and hydrophobic material properties, has been shown to be successful. In particular, the technique enables the affordable upscaling of microfluidic biofuel cells through 3D printing. In general, the technique would be of high utility to any application that simultaneously requires distributed wiring and fluidic access inside 3D printed devices.

## Data Availability

Not applicable.
